# Effect of Neodymium-Doped Yttrium Aluminum Garnet (Nd:YAG) Laser Enamel Pre-Treatment on the Whitening Efficacy of a Bleaching Agent

**DOI:** 10.7759/cureus.31325

**Published:** 2022-11-10

**Authors:** Vaishnavi S Borse, Varsha Sanjay Pandit, Ashwini Gaikwad, Abhijit Bajirao Jadhav, Aishwarya Handa, Ruchira Bhamare

**Affiliations:** 1 Department of Conservative Dentistry and Endodontics, Bharati Vidyapeeth Dental College and Hospital, Pune, IND

**Keywords:** whitening, spectrophotometer, laser, color analysis, bleaching

## Abstract

Background: There are a number of different substances and treatments that are effective in mitigating the negative effects of tooth bleaching. It is essential, however, to consider whether or not the presence of these factors affects the efficiency of the bleaching agent.

Aim: The purpose of this study is to determine how the pretreated enamel with a Neodymium-Doped Yttrium Aluminum Garnet (Nd:YAG) laser affects the bleaching effect of hydrogen peroxide at a concentration of 35%.

Methods and materials: Thirty samples were prepared from human-extracted permanent anterior teeth and stained using a tea solution. Based on the pre-treatment protocol, the samples were split into two equal groups (n = 15): In Group I, samples were submerged in saline solution for five minutes, while in Group II, Nd:YAG laser treatment was performed. Following pretreatment, samples from both groups were bleached with 35% hydrogen peroxide. Colour analysis of all samples was done before and after bleaching using a spectrophotometer. For the colour analysis, the CIE L*a*b* (Commission Internationale de l'Eclairage) System of colour measurement was used. For statistical analysis, Student t-tests (two-tailed, unpaired) were used to compare the means of two groups on a continuous scale.

Results: Samples from both groups became lighter in colour following bleaching. After bleaching, there was no discernible difference in the total colour change between the two groups.

Conclusion: The application of the Nd:YAG laser before bleaching did not influence the whitening efficacy of 35% hydrogen peroxide.

## Introduction

In-office bleaching with hydrogen peroxide is extremely sought after due to excellent tooth-whitening results. However, hydrogen peroxide has the potential to cause undesirable effects that include dentin hypersensitivity, cervical resorption, and alterations of the tooth surface [1]. To counteract these adverse effects, various re-mineralizing agents such as sodium fluoride, nano-hydroxyapatite, casein phosphopeptide-amorphous calcium phosphate (CPP-ACP), and lasers have been tried out successfully, either before or after the bleaching procedure [2-4]. When applied to enamel either before or after bleaching, lasers have demonstrated reduced stain absorption and increased micro-hardness of enamel, compared to bleached enamel not subjected to laser [5].

However, it is essential to take into consideration whether or not any of the remineralizing agents have a negative impact on the whitening or bleaching effectiveness of the bleaching agent. If the remineralizing agent makes the bleaching treatment less effective, then the results that are produced from the bleaching procedure will not be as good as they possibly are. In the past, it was seen that the application of sodium fluoride before bleaching did not influence the whitening efficacy of the bleaching agent [6], but a major limitation of topical fluorides is that their effects are short-lasting and require frequent re-applications due to unpredictable diffusion. whereas lasers have demonstrated effective, long-lasting results after a single application. Due to this advantage, lasers are becoming increasingly popular, especially for treating dentin hypersensitivity [7,8].

As a result, we have made an effort to determine whether or not the use of lasers prior to the application of a bleaching agent has the potential to alter the level of whitening achieved by the agent. The objective of this study was to determine if enamel pre-treatment with Nd:YAG laser has any influence on the whitening efficacy of 35% hydrogen peroxide. The findings of our research will assist us in determining whether or not lasers may be effectively used to mitigate the unfavourable effects of bleaching without having a detrimental impact on the efficiency of the bleaching process.

## Materials and methods

Thirty extracted human permanent anterior teeth were collected which were extracted at another department for particular reasons. Teeth with visible surface defects such as wear, cracks, caries, restorations or discolourations due to developmental disturbances were excluded. The teeth were thoroughly cleaned and disinfected.

The middle third of the labial surface of the tooth was used for colour analysis. The working area was carved by four marks, corresponding to the corners of an imaginary square in the middle third of the labial surface of each tooth, using a round carbide bur of diameter 0.7 mm (MANI, Tochigi, Japan) and a high-speed handpiece (Nakanishi Inc., Tochigi, Japan).

Tooth samples were then subjected to a uniform staining procedure using a tea solution. It was prepared by immersing two tea bags containing 2.5 g of 98% black tea (Marks and Spencers) in 100 ml of boiling water for five minutes. During the study, samples were kept in a tea solution for six days at room temperature. After that, they were stored in distilled water.

The colour of the uniformly stained samples served as the baseline colour against which future colour comparisons were made. Color analysis for each sample was performed using a UV Visible Spectrophotometer (JASCO-V-770 UV-Visible/NIR). The sample was positioned such that the incident beam of light fell onto the middle third of the labial surface.

The colour of the sample was measured using the CIE L*a*b* (Commission Internationale de l'Eclairage) colour space. In a spectrophotometer, a picture of the sample is automatically translated into a series of numerical values expressed in the L*a*b* system. The L* axis represents the degree of lightness and ranges from 0 (black) to 100 (white). The a* plane represents the degree of green/red colour, while the b* plane represents the blue/yellow colour. Three baseline colour readings were recorded for every sample, and their average served as the baseline colour reading/value for that particular sample. To standardise the baseline colour of all sample, 30 samples that demonstrated an L* value between 40 and 50 were selected. The samples were then randomly assigned to two experimental groups (n = 15) as follows: Group I: Control group: Samples in this group were immersed in 0.9% normal saline for 5 minutes before a common bleaching procedure. Group II: Samples in this group were subjected to the Nd:YAG laser before a common bleaching procedure. The Nd:YAG laser (AT S LightWalker®; Fotona, Ljubljana, Slovenia) was utilised in a non-contact continuous mode, at an energy of 1 W, 15 Hz for 60 seconds, twice. The laser tip was held at a distance of approximately 1 cm from the sample.

After 24 hours of pre-treatment protocol, samples from both groups were subjected to a common bleaching procedure using 35% hydrogen peroxide (Pola Office; SDI, Victoria, Australia). The middle one-third of the tooth's labial surface was treated with the bleaching agent, as recommended by the manufacturer. The bleaching was done for 16 minutes, during which the bleaching agent was light-activated with a LED (light emitting diode) curing lamp (Walden, Smart) only once for 40 seconds. After bleaching, samples were subjected to colour analysis, as described previously. 

Color measurements of the bleached samples were compared with their baseline, to find the total color change (ΔE). The differences in L*, a*, and b* values between baseline and after bleaching were calculated and expressed as ΔL, Δa, and Δb, respectively for each sample. The following formula was used to determine the total colour difference ΔE for each sample:h

ΔE = (ΔL^2^+Δa^2^+Δb^2^)^1/2^.

## Results

Color analysis in accordance with the CIE L*a*b* system was done for Group I and Group II, before and after bleaching. Table 1 depicts the mean/average L*a*b* value for each group, before (baseline) and after the bleaching (final) procedure.

**Table 1:** Color analysis before and after bleaching for Group I and Group II according to the CIE L*a*b* system of color measurement

Groups	Mean Baseline Value			Mean Final Value			Mean Colour Difference (Δ)			
	L*	a*	b*	L*	a*	b*	ΔL	Δa	Δb	ΔE
Group I	46.455	2.522	12.765	58.599	2.190	10.661	12.145	-0.332	-2.104	12.358
Group II	46.615	2.709	13.368	59.013	1.845	10.615	12.397	-0.865	-2.753	12.739

The mean Δ L*, Δ a*, Δ b*, and Δ E have been calculated for each group. The increase in L* value and decrease in a* and b* values after bleaching for tooth samples in both groups indicates that all the tooth samples had become lighter following bleaching. Table 2 compares Delta E values in terms of Mean (SD) across both groups using an unpaired t-test.

**Table 2:** Comparison of Δ E values in terms of (Mean {SD}) among both the groups using unpaired t-test

Groups	N	Mean ΔE	Standard Deviation	t value	P value
Group I	15	12.35813	1.425111	0.700	0.489
Group II	15	12.73893	1.550418		

There was statistically no significant difference between the two groups concerned as the p-value obtained was greater than 0.05. This implies that the bleaching efficacy for both groups was similar.

## Discussion

Dentin hypersensitivity, surface roughness, stain absorption, and diminished micro-hardness are common following bleaching [1,9]. Lasers may induce tooth surface melting by fusing and recrystallizing hydroxyapatite crystals. This creates a smooth, micro-hardened tooth surface. The smoother surface reduces post-operative staining [6,10]. The hydroxyapatite crystal fusion and recrystallization occlude dentinal tubules, reducing hypersensitivity [11,12]. Protein precipitation in dentinal fluid and a photo-modulating action that stimulates tertiary dentin development may also ease dentin hypersensitivity [12].

Almost all topical desensitizers have a delayed start of the action and a short-lasting effect for dentin hypersensitivity. Lasers have proven good benefits after only one treatment, with rapid pain alleviation. This is because they immediately narrow and occlude dentinal tubules. Laser desensitizers are common [3,8,13,14].

However, it is important to note if the agent used to counteract the negative effects of bleaching interferes with the bleaching agent's whitening efficacy. Hence, we have tried to evaluate if the pre-operative use of lasers can affect the whitening efficacy of a bleaching agent. In-vitro studies that assess the bleaching effect on teeth may involve sectioning or polishing teeth [6] or may not involve any alterations made to them [5]. In clinical conditions, teeth to be bleached are never subjected to sectioning or polishing. Hence, we did not subject the teeth to any alterations. To define our working area, four marks, corresponding to an imaginary square, were carved in the middle third of the labial surface of each tooth in accordance with a previous study [15].

In order to appreciate the colour difference after bleaching, all teeth were subjected to a uniform staining procedure using a tea solution, as per the protocol suggested by previous studies [5,16]. The tea solution leads to more intrinsic discoloration than extrinsic, which is ideal for assessing the effects of bleaching. A six-day period of immersion in tea solution was followed as it has been seen that maximum tooth discoloration occurs during the first 24 hours and a minimum of five days [16].

Intermediate storage of tooth samples was done in distilled water as we did not want to take into account the remineralization effect that saliva might have on the tooth. It has been seen that there might be a variation in the remineralization and optical properties of teeth, depending upon the type of saliva (natural or artificial) used as the storage medium [17]. Commonly used methods for colour assessment of teeth are visual, spectrophotometric, and colorimetric. Most studies state that spectrophotometric colour determination is the most accurate, reliable, and reproducible method [18-20].

Different lasers used for remineralization and treating dentin hypersensitivity include a diode, carbon dioxide, Nd:YAG, Er:YAG, and ErCr:YSGG lasers [12,21]. We chose the ND:YAG laser, as it has fewer chances of crack propagation in teeth when compared to most of the other lasers and is easily available [18,21]. The Nd:YAG laser is commonly operated at a power output of 0.3 to 2 W in a continuous or pulsed mode for dentin hypersensitivity and remineralization [12,21]. The protocol for the application of the ND:YAG laser in the present study was in accordance with a previous study [22].

Following bleaching, it was observed that samples in both the groups had become lighter, as there was a shift of the L* value towards the positive side (lighter) and the b* value towards the negative side (less yellow and more blue). For the human eye to detect a colour change, ΔE should be greater than or at least equal to 1 [23]. In the present study, ΔE for both the groups was approximately 12, indicating that visible colour difference had occurred after bleaching in both groups. There was statistically no significant colour difference (ΔE) between the laser and the control group. This implied that the whitening efficacy of the bleaching agent was unaffected by Nd:YAG laser pre-treatment. This finding is in agreement with a previously done study in which a combination of fluoride and Nd:YAG laser application was done before bleaching and their effect on colour change ΔE and luminosity ΔL was evaluated. There was statistically no significant difference between two groups in their study as far as total colour change and luminosity are concerned [18].

It was feared that the penetration of the bleaching agent into the tooth structure might be hampered due to the action of the laser, which involves hydroxyapatite recrystallization and subsequent occlusion of tubules. However, in this study, it was observed that the bleaching efficacy was unaffected in spite of laser pre-treatment. A possible reason could be, that dental hard tissues are highly permeable to fluids, and fluid flow in the enamel and dentin occurs through inter-prismatic spaces and dentinal tubules, respectively. Therefore, hydrogen peroxide, due to its low molecular mass, can easily diffuse through teeth [24]. Besides this, although lasers can cause tooth surface modifications, they do not completely alter the tooth surface [21]. We assume that the ability of hydrogen peroxide to penetrate into the tooth remains the same since not all the porosities in the tooth surface are eliminated.

In the future, similar studies can take into consideration the effect of saliva and other lasers at different power settings. Also, variations in the bleaching protocol can be done.

## Conclusions

From the results and within the limitations of this in-vitro study, it can be concluded that the application of Nd:YAG laser before bleaching, does not have any influence on the bleaching/whitening efficacy of 35% hydrogen peroxide. There was statistically, no significant color difference (ΔE) between the tooth samples belonging to Group I and Group II following bleaching, which indicated that the bleaching efficacy in both groups was similar.

Since laser pre-treatment did not have any influence on the whitening efficacy of a bleaching agent, we can consider laser pre-treatment to counteract the adverse effects of bleaching as an acceptable treatment option. However, we recommend further studies be carried out with changes in the type or mode of application of lasers or bleaching agents, prior to their clinical implementation.
